# Targeting of client proteins to the VCP/p97/Cdc48 unfolding machine

**DOI:** 10.3389/fmolb.2023.1142989

**Published:** 2023-02-07

**Authors:** Hemmo Meyer, Johannes van den Boom

**Affiliations:** Center of Medical Biotechnology, Faculty of Biology, University of Duisburg-Essen, Essen, Germany

**Keywords:** protein homeostasis, ubiquitin, protein unfolding, protein phosphatase (PP) 1, protein quality control, DNA replication and damaged repair

## Abstract

The AAA+ ATPase p97 (also called VCP or Cdc48) is a major protein unfolding machine with hundreds of clients in diverse cellular pathways that are critical for cell homeostasis, proliferation and signaling. In this review, we summarize recent advances in understanding how diverse client proteins are targeted to the p97 machine to facilitate client degradation or to strip clients from binding partners for regulation. We describe an elaborate system that is governed by at least two types of alternative adapters. The Ufd1-Npl4 adapter along with accessory adapters targets ubiquitylated clients in the majority of pathways and uses ubiquitin as a universal unfolding tag. In contrast, the family of SEP-domain adapters such as p37 can target clients directly to p97 in a ubiquitin-independent manner. Despite the different targeting strategies, both pathways converge by inserting the client into the p97 pore to initiate a peptide threading mechanism through the central channel of p97 that drives client protein unfolding, protein extraction from membranes and protein complex disassembly processes.

## Introduction

Proteins need to fold into a three-dimensional structure to become functional. Conversely a large fraction of these proteins have to be actively unfolded at least once in their lifetime for regulation or to assist their degradation. Protein unfolding is usually mediated by a family of ATPases Associated with diverse Activities (AAA) proteins (Erzberger and Berger, 2006; Khan et al., 2022). The most abundant and functionally versatile AAA protein is p97 (also called VCP or Cdc48). p97 has a key role in the ubiquitin-proteasome system and thus for protein homeostasis as it processes misfolded and ubiquitylated proteins to prepare them for degradation in the 26S proteasome in diverse pathways including ER-associated degradation (van den Boom and Meyer, 2018; Stach and Freemont, 2017; Ye et al., 2017). Moreover, p97 helps degradation of regulatory proteins for terminal inactivation in a number of signaling pathways. While the 26S proteasome has its own AAA protein ring, p97 is needed for clients that require extraction from membranes or partner proteins, but also for a subpopulation of monomeric proteins to provide unfolded peptide stretches that are essential for processing by the proteasome (Beskow et al., 2009; van den Boom and Meyer, 2018; Olszewski et al., 2019). In addition to its degradative function, p97 mediates regulatory unfolding and protein complex disassembly for example during protein phosphatase-1 (PP1) biogenesis (Weith et al., 2018). Missense mutations in p97 cause a multisystem proteinopathy (MSP-1) with features such as inclusion body myopathy, Paget’s disease of bone, frontotemporal dementia and amyotrophic lateral sclerosis (Al-Obeidi et al., 2018; Watts et al., 2004; Meyer and Weihl, 2014). Conversely, p97 is considered a promising cancer drug target (Anderson et al., 2015; Roux et al., 2021).

**FIGURE 1:**
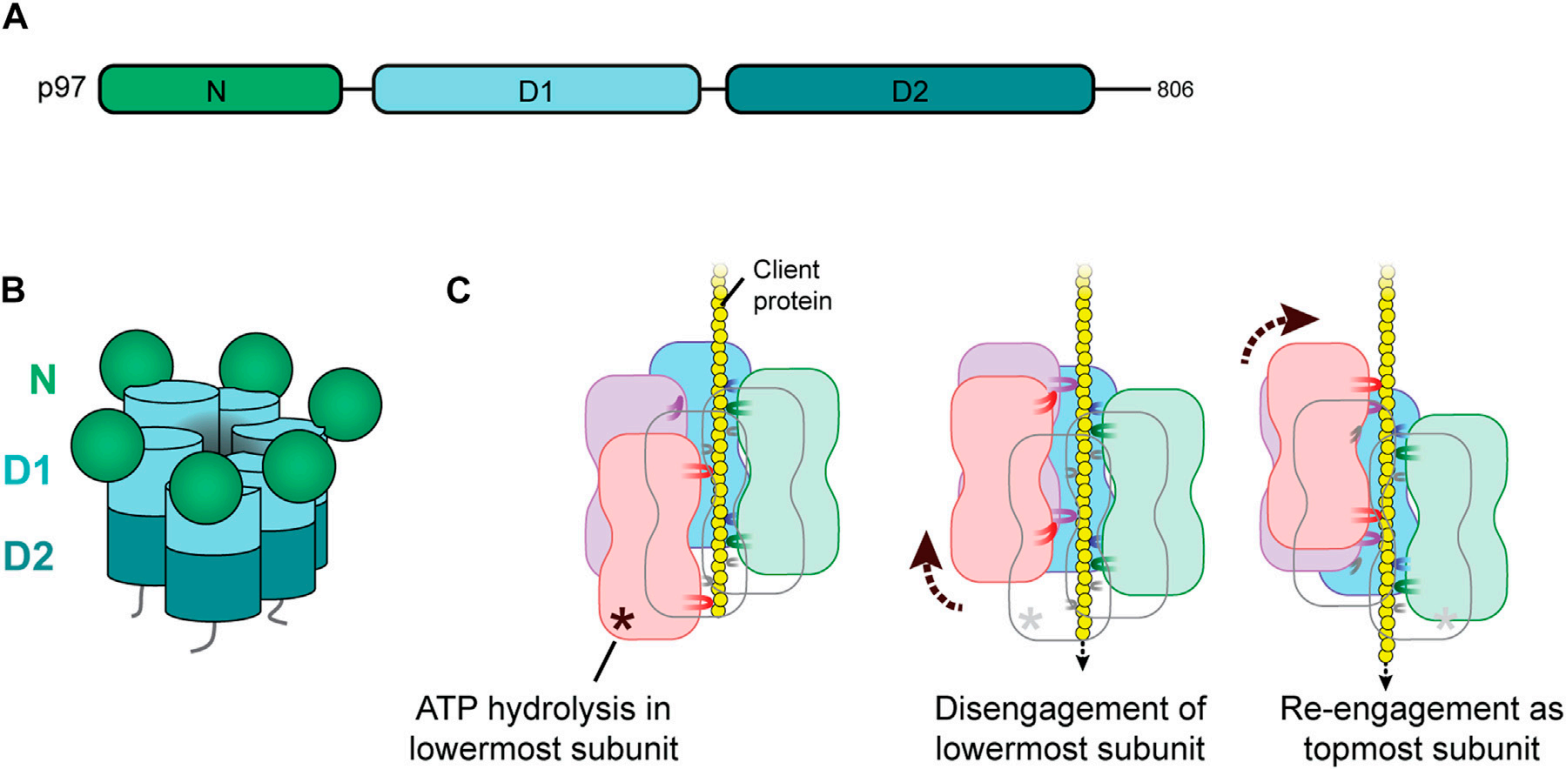
Structure and client threading mechanism of p97. **(A)** Domain structure of p97. Each p97 subunit comprises an N-terminal domain and two AAA ATPase domains, D1 and D2. **(B)** Cartoon representation of the p97 hexamer in staircase configuration around the central channel. **(C)** Hand-over-hand mechanism for client threading by p97. When the client protein (yellow) is inserted into the central channel of p97, hydrophobic pore loops of D1 and D2 interact with the client backbone. Each subunit binds two residues below the previous subunit (in clockwise direction) which sets the p97 hexamer in the right-handed staircase configuration. ATP hydrolysis in the lowermost subunit (orange) triggers the retraction of its pore loops and disengagement of the subunit (middle panel) creating a seam in the hexamer. Subsequent ADP release and re-binding of ATP induces re-engagement of this subunit as the topmost subunit (right panel). Propagation of ATP hydrolysis and up-movement of subunits around the hexamer in counterclockwise direction threads the client through the channel.

p97 has two AAA domains, D1 and D2 that form two stacked hexameric rings with a central channel (Figures 1A, B). The regulatory N-terminal domain is positioned at the periphery of the D1 ring and can be in a up or down position. Recent structural and biochemical work has revealed that client proteins are unfolded, or stripped from partners or membranes, by inserting them into the D1 pore and threading them through the central channel followed by ejection from the D2 pore (Bodnar and Rapoport, 2017; Weith et al., 2018; van den Boom et al., 2021). The active hexamer is in a stair-case configuration that allows a client threading mechanism that is common to many AAA unfoldases (Cooney et al., 2019; Twomey et al., 2019; Stach et al., 2020; Pan et al., 2021a; van den Boom et al., 2022; Xu et al., 2022). Hydrophobic residues in the pore loops engage in non-sequence-specific interactions with the client peptide backbone (Figure 1C). Threading is driven by a hand-over-hand mechanism in which ATP hydrolysis in the subunit at the bottom of the p97 spiral results in a discontinuity as it triggers the detachment of this subunit from the spiral and induces its reattachment to the client peptide at the top. Thus, the peptide is pulled through the pore with the progression of nucleotide binding and hydrolysis around the ring.

To achieve its versatility, p97 is assisted by a host of cofactor proteins that directly bind to p97 through dedicated interaction motifs and domains (Meyer and Weihl, 2014; Buchberger et al., 2015; Stach and Freemont, 2017). These cofactor proteins include client adapters, accessory adapters, membrane recruitment factors and client processing enzymes. A large body of cell biological and genetic data is available on the functions of these cofactors in the cell. Direct mechanistic analysis, however, has only become possible recently with the reconstitution of client unfolding *in vitro*. Key technical advances were the efficient *in vitro* ubiquitylation of a model client for ubiquitin-directed targeting and the discovery of a PP1 complex as a ubiquitin-independent client of p97 (Blythe et al., 2017; Bodnar and Rapoport, 2017; Weith et al., 2018; Mukherjee and Labib, 2019). This was combined with the use of GFP and later Eos fused to the clients, as well as the development of a FRET assay that monitor the unfolding or disassembly of clients, respectively, in real time (Blythe et al., 2017; Bodnar and Rapoport, 2017; Weith et al., 2018; Olszewski et al., 2019; van den Boom et al., 2021). Insertion and threading of clients in the p97 channel has been observed by cryo-EM, but can also be demonstrated by genetically encoded photocrosslinkers placed in the p97 pore loops and other critical positions (Bodnar and Rapoport, 2017; Weith et al., 2018). Thus, the field now profits from a powerful biochemical toolbox for dissecting and understanding the mechanism of client targeting to p97.

In this article, we will therefore focus on factors that have a biochemically proven function in the targeting of client proteins to the p97 channel and will only discuss a few additional candidates. For a more comprehensive discussion of p97 cofactors, we refer the reader to previous reviews (Meyer and Weihl, 2014; Buchberger et al., 2015; Stach and Freemont, 2017).

## Two alternative pathways of targeting through distinct adapters

**FIGURE 2:**
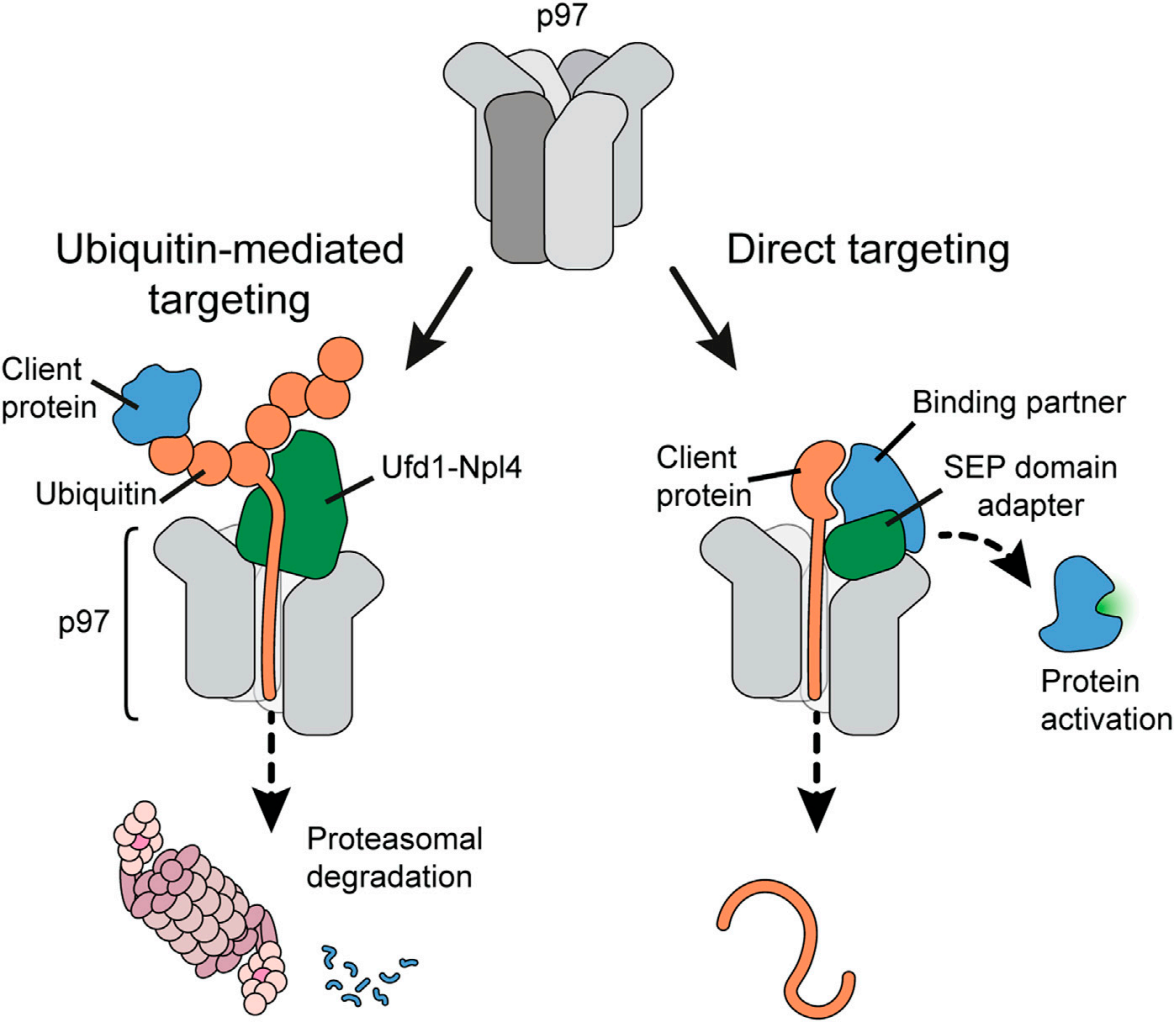
Client targeting pathways to p97. p97 uses (at least) two alternative strategies for client protein targeting. Ubiquitin-mediated targeting (left panel) recruits ubiquitylated client proteins to p97 using ubiquitin as a universal targeting tag. Ubiquitin chains attached to the client are bound by the Ufd1-Npl4 adapter. One ubiquitin is then melted and inserted by the N-terminus into the p97 pore. Continuous threading of the ubiquitin chain eventually leads to threading and unfolding of the client protein, often for subsequent degradation in the proteasome. In contrast, direct client targeting (right panel) uses adapter proteins of the SEP domain family to guide a p97 targeting region within the client into the p97 pore. Pulling the client protein through the p97 pore dislodges the protein from its binding partner, which can result in activation of the binding partner.

An important question has been how client proteins are recruited to p97 and, importantly, guided into the D1 pore to initiate client peptide threading. The molecular challenge is on the one hand to target a large diversity of clients for degradative unfolding, and on the other hand be selective for a direct target while sparing binding partners during regulatory protein complex disassembly. Early work had already suggested that p97 is directed by alternative, mutually exclusive client adapter proteins: the heterodimer Ufd1-Npl4 or members of a family of SEP-domain adapters (Figure 2) (Kondo et al., 1997; Meyer et al., 2000; Buchberger, 2022). Although structurally very different, both types of adapters bind p97 through a bipartite interaction mechanism. Ufd1-Npl4 appears to handle the majority of ubiquitin-modified clients, often to facilitate degradation in the proteasome. Ufd1-Npl4 is therefore crucial for pathways such as ER-associated degradation or ribosomal quality control as well as DNA-associated processes such as DNA repair and replication. In contrast, SEP-domain adapters such as p37 can mediate a selective, ubiquitin-independent targeting mechanism that discriminates a direct target in protein complex disassembly. With about 230,000 p97 hexamers and 70,000 copies of Ufd1-Npl4 in an exemplary human cancer cell (Beck et al., 2011), roughly one third of the p97 hexamers could be equipped with Ufd1-Npl4 at a time. In the same cell, more than 285,000 copies of SEP domain adapters exist (Figure 4A). However, not all p97 hexamers are thought to be stably occupied by adapters (Xue et al., 2016).

### The Ufd1-Npl4 pathway targets ubiquitin chains as a universal unfolding tag

**FIGURE 3:**
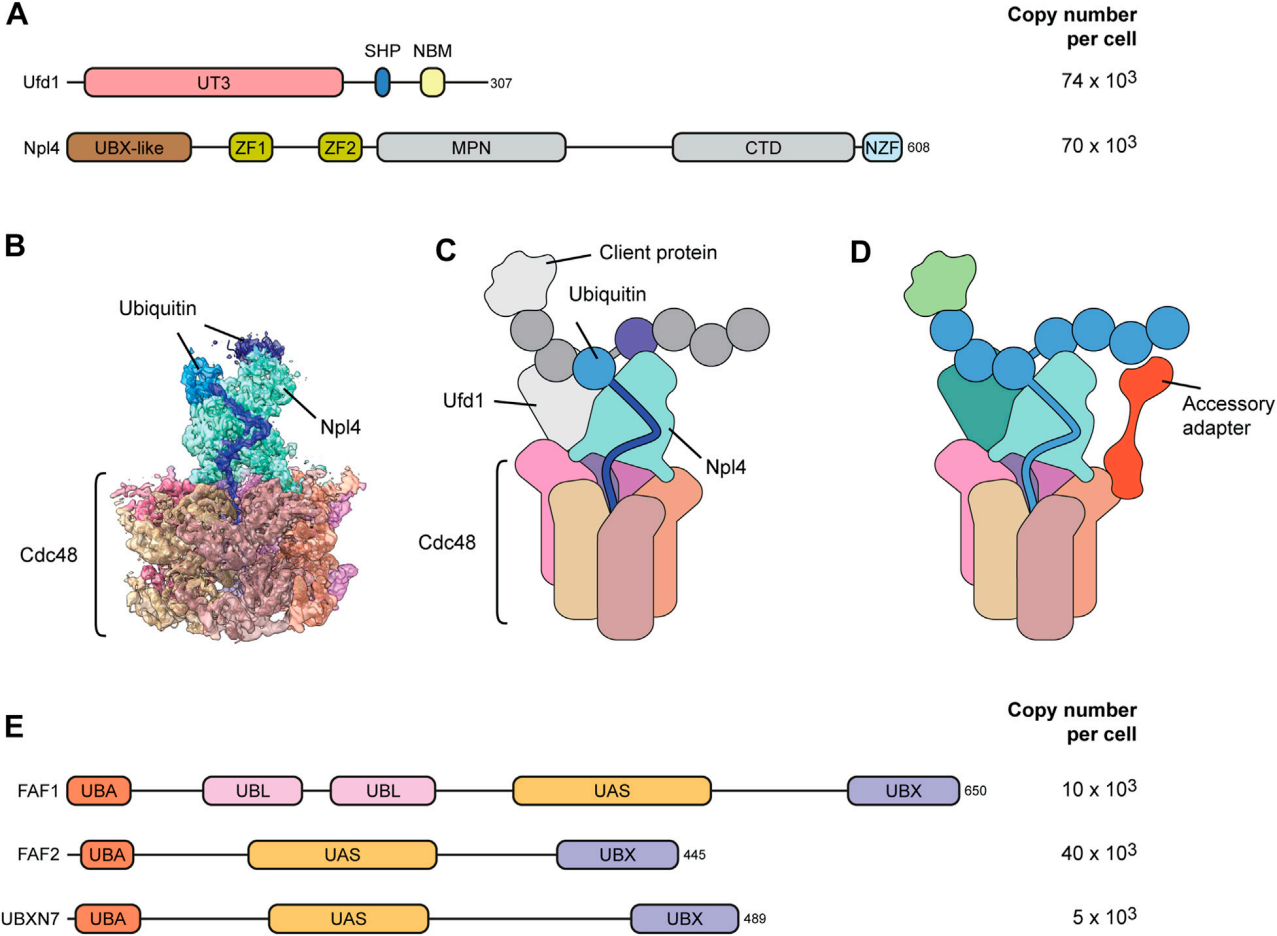
Adapter and accessory adapters for ubiqutin-mediated targeting. **(A)** Domain structure of the major Ufd1-Npl4 client adapter. Ufd1-Npl4 is a heterodimer that binds p97 cooperatively *via* interactions of a UBX-like (UBXL) domain in Npl4 and a SHP box in Ufd1 with p97 N-domains. The zinc fingers (ZF1 and ZF2) in Npl4 make contact with the top of the D1 ring. The UT3 domain in Ufd1 and the C-terminal domain (CTD) in Npl4 bind and position the ubiquitin chain. The ubiquitin-binding NZF is specific for metazoan Npl4. NBM, Npl4 binding motif. MPN, Mpr1, and Pad1 N-terminal domain. Copy numbers of indicated proteins compared to roughly 230 × 10^3^ p97 hexamers in human U2OS osteosarcoma cells according to (Beck et al., 2011). **(B)** Cryo-EM structure (pdb 6OA9 and EMD-0665) of yeast Cdc48-Ufd1-Npl4 with a ubiquitinated model substrate in the central pore. Dark blue depicts one unfolded ubiquitin moiety bound to a groove in Npl4 (cyan). Additional ubiquitin moieties are bound on top. **(C)** Cartoon model of the structure in **(B)**. Ufd1, the client and additional ubiquitin moieties are not resolved in the structure. **(D)** Speculative model for the function of accessory adapters such as FAF1, FAF2 or UBXN7. Accessory adapters enhance p97 affinity for the client by bridging p97 with the ubiquitin chain attached to the client, thereby assisting Ufd1-Npl4-mediated client targeting and unfolding. **(E)** Domain structure of p97 cofactor proteins that have been proven to act as accessory adapters cooperating with Ufd1-Npl4. They are characterized by UBX and UBA domains that bind p97 and ubiquitin, respectively, and possess a thioredoxin-like (UAS) domain of yet unknown function. UBL, ubiquitin-like domain.

The Ufd1-Npl4 adapter is a heterodimer that cooperatively binds p97 through a SHP box in Ufd1 and a UBX-like domain in Npl4 (Figure 3A) (Bruderer et al., 2004). Npl4 does not bind p97 in the absence of Ufd1, and Ufd1 is destabilized upon Npl4 depletion suggesting that they always act together (Meyer et al., 2000; Wojcik et al., 2004). Recent cryo-EM structures of Ufd1-Npl4 with p97 and a ubiquitylated client protein revealed how Ufd1-Npl4 recruits clients and initiates their threading through the p97 channel (Twomey et al., 2019; Pan et al., 2021a; Pan et al., 2021b); (Figure 3B). Npl4 forms a tower above the D1 pore with two zinc binding domains resting on the D1 ring and the UBX-like domain interacting with one N-domain of p97 (Figures 3B, C). Ufd1 is poorly resolved in the published structures, but it binds to the N-domain of p97, opposite to the Npl4-bound N-domain, thereby positioning the Npl4 tower above the p97 pore.

Rather than binding the client directly, Npl4 interacts with the ubiquitin chain attached to the client (Twomey et al., 2019; Pan et al., 2021a; Sato et al., 2019; Pan et al., 2021b); (Figures 3B, C). The interaction is mediated by several moieties of the ubiquitin chain binding the top of the Npl4 tower. Importantly, one of the ubiquitin moieties is unfolded by extending into a groove in Npl4 that guides the elongated peptide stretch towards the p97 D1 pore and inserts it into the p97 channel (Twomey et al., 2019). p97 thereby first threads the ubiquitin chain and subsequently pulls in the attached client leading to unfolding of the client. This elegant strategy uses the ubiquitin modification as a universal unfolding tag that is independent of the client protein which it is conjugated to. This is very different from the regulatory particle of the proteasome which recruits the client through binding of the attached ubiquitin chain, but then requires an unfolded stretch of the client to insert the client directly into the AAA ring for unfolding while the ubiquitin chain is clipped off (Greene et al., 2020). This difference explains why some clients that do not have an unfolded peptide stretch require prior unfolding by p97 to facilitate processing and degradation by the proteasome (Beskow et al., 2009; Olszewski et al., 2019).

Starting the pulling on one of the ubiquitin moieties, however, also entails that the threading machine will soon encounter the branch point within ubiquitin chains and eventually the attachment site of the ubiquitin to the client (Twomey et al., 2019). Initial data indicated a requirement of a deubiquitinating enzyme that removes at least the distal parts of the ubiquitin chain (Bodnar and Rapoport, 2017). However, unfolding can occur in the absence of deubiquitinating enzymes (Olszewski et al., 2019) suggesting that p97 needs to be able to thread branch points and loops as shown by Ji and collegues (Ji et al., 2021). In support of that, independent data indicate that p97 can transport peptide loops (see below) and even peptides attached to oligonucleotides indicating a degree of plasticity regarding the structures that are threaded in the p97 channel (van den Boom et al., 2021; Kroning et al., 2022). An interesting question remains whether p97 can pull on only one strand when threading a loop, or on both strands as shown for the related AAA protein ClpB (Avellaneda et al., 2020). The function of Ufd1 in client targeting has not been uncovered yet. Intriguingly the globular UT3 domain of Ufd1 has the same fold as the p97 N-domain suggesting it could assist binding or unfolding the client.

### Ufd1-Npl4 cooperates with accessory adapters

*In vitro*, the Ufd1-Npl4 adapter is sufficient to trigger p97-driven unfolding of ubiquitylated clients. However, a growing body of evidence has been indicating that Ufd1-Npl4 cooperates with a family of accessory adapters comprising FAF1, FAF2 and UBXN7 during client targeting in the cell (Figures 3D, E). These accessory adapters are less abundant than Ufd1-Npl4 (Figure 3E) suggesting that they cooperate with Ufd1-Npl4 as needed. Like many p97 cofactors they contain a ubiquitin-binding UBA and a p97-interacting UBX domain at the N and C-terminus, respectively. In addition, the accessory adapters are characterized by a thioredoxin-like UAS domain of yet unknown function. Early data showed that yeast Ubx2 and its mammalian counterpart FAF2 (also called UBXD8) serves as targeting factor during p97-Ufd1-Npl4 mediated ER-associated degradation (Neuber et al., 2005; Schuberth and Buchberger, 2005). Of note, Ubx2 and FAF2 insert into the cytosolic leaflet of the ER membrane *via* a hydrophobic hairpin loop and can also partition into the mitochondrial membrane where it assists p97-Ufd1-Npl4 in mitochondria-associated degradation (Metzger et al., 2020). In contrast, FAF1 does not have a yeast orthologue. FAF1 (UBXN-3 in *C. elegans*) is distributed in both the cytosol and nucleoplasm but has functionally been mostly associated with various DNA-associated roles of p97-Ufd1-Npl4 such as degradation of the licensing factor CDT1 or extraction of topologically trapped DNA repair factor KU70/80 extraction (Franz et al., 2016; van den Boom et al., 2016). The third member, UBXN7 (Ubx5 in yeast), is localized exclusively in the nucleus and has been linked to various chromatin-associated functions of p97 (Alexandru et al., 2008; Verma et al., 2011; Puumalainen et al., 2014; Chauhan et al., 2021). Both FAF1 and UBXN7 have been connected to the extraction of the replicative helicase from DNA (Sonneville et al., 2017; Xia et al., 2021; Kochenova et al., 2022). The replicative helicase forms a ring consisting of the AAA proteins MCM2-7 and is assembled tightly around DNA during replication origin licensing. Consequently, the MCM2-7 ring needs to be actively dissociated at termination of replication or when the helicase encounters obstacles such at interstrand crosslinks. Disassembly is triggered by ubiquitylation of MCM7 which is then targeted and extracted by p97 and Ufd1-Npl4 leading to destabilization of the whole complex. Crucially, UBXN7 assists p97-Ufd1-Npl4-mediated extraction of the helicase (Sonneville et al., 2017; Xia et al., 2021; Kochenova et al., 2022). FAF1 can compensate for UBXN7, although this is controversial (Fujisawa et al., 2022; Tarcan et al., 2022).

Recent advances in reconstituting MCM2-7 disassembly from pure components have confirmed the involvement of the accessory adapters and brought more clarity in the molecular basis (Fujisawa et al., 2022). p97-Ufd1-Npl4 suffices to disassemble ubiquitylated MCM2-7 but only if the ubiquitin chains are very long. If ubiquitin chains are shorter with at least five ubiquitin moieties, p97-Ufd1-Npl4 is much less efficient but can be stimulated by any of the three factors. Surprisingly, truncation analysis revealed that the stimulatory effect does not depend on the UBA domain, at least for FAF1. In fact, apart from the UBX domain in FAF1, a helical domain located between the UAS and UBX domains was sufficient to stimulate disassembly of complexes modified with short ubiquitin chains. So far, it is unclear what this region binds to in the client. The authors conclude that a threshold for the ubiquitin chain length exists that can be overcome with the accessory adapters (Fujisawa et al., 2022). Since the analysis was done only with endpoint measurements it will be interesting to clarify whether the accessory adapters have a more general effect on the rate of client processing. Analysis of disassembly rates would also allow a more detailed dissection of the contribution of the different domains.

### SEP-domain adapters can target clients directly in a ubiquitin-independent manner

**FIGURE 4:**
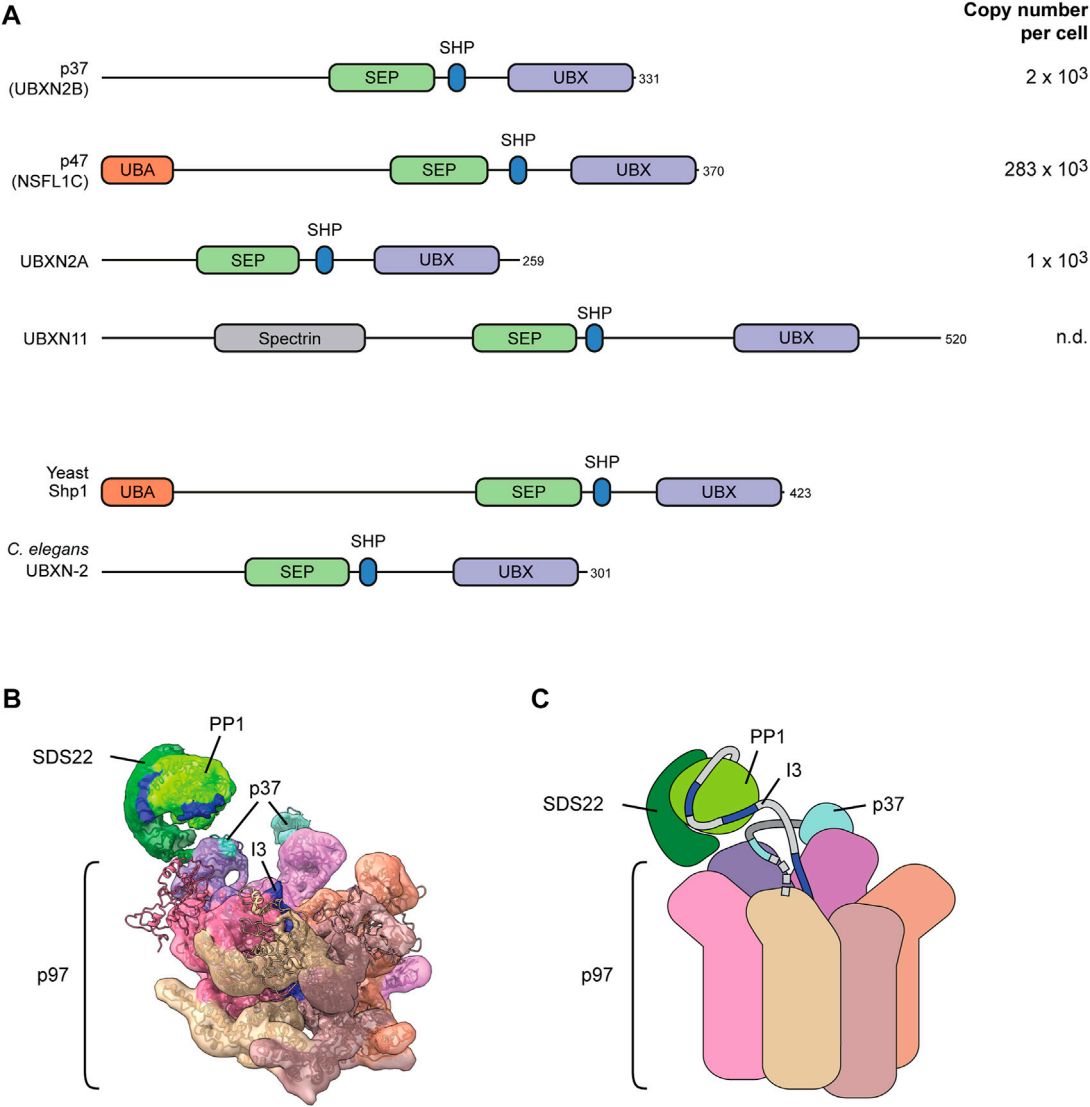
The SEP-domain adapters for direct client targeting. **(A)** Domain structure of the four human SEP domain proteins. Yeast and *C. elegans* code for only one ortholog as depicted. The UBX domain and the SHP box motif mediate binding to the p97 N-domain. The defining SEP domain contributes to a multivalent substrate binding mechanism. The UBA domain binds ubiquitin. Copy numbers of proteins in human U2OS osteosarcoma cells according to (Beck et al., 2011). n.d., not detected. **(B)** Cryo-EM structure of p97-p37-SDS22-PP1-I3. Dark blue indicates sections of I3 inserted into the p97 while still associated with PP1 (bright green). Note direct interaction of SDS22 (dark green) with an N-domain (purple) of p97. The SHP box and UBX domain of p37 (cyan) bridge two adjacent N-domains. **(C)** Cartoon model of the structure in **(B)**. Dashed line indicates the N-terminal portion of p37 that is not resolved and includes the SEP domain.

The alternative family of adapters is defined by the SEP (**S**hp1, **e**yes-closed, **p**47) domain combined with the p97-interaction module consisting of a SHP box and a UBX domain. Four SEP-domain proteins are encoded in the human genome (Figure 4A). p37 (also called UBXN2B), UBXN2A and UBXN11 do not contain ubiquitin-binding elements. p47 (also called NSFL1C) is an exception and harbors a ubiquitin-binding UBA domain. Whereas *S. cerevisiae* has only one SEP-domain protein, Shp1, with a UBA domain, the only orthologue in *C. elegans* lacks the UBA domain (Figure 4A).

Earlier work showed differential involvement of ubiquitin in the functions of p47 and p37 in SNARE-mediated mitotic membrane dynamics (Meyer, 2005; Totsukawa et al., 2011). The recently uncovered regulation of PP1 biogenesis by p97 and its SEP-domain adapters has brought clarity to the mechanism of client targeting and protein complex disassembly. Work in yeast already demonstrated that Shp1 (**S**uppressor of **h**igh copy **P**P1) is needed for activation of PP1 (rather than for its degradation) (Zhang et al., 1995). Further analysis in the mammalian system showed that newly synthesized PP1 is first held in an intermediate complex with its partners SDS22 (also called PPP1R7) and inhibitor-3 (I3, also called PPP1R11) to keep PP1 inactive (Weith et al., 2018; Cao et al., 2021). The SDS22-PP1-I3 complex is very stable and needs to be dissociated by p97 to allow assembly of PP1 holoenzymes with activating subunits (Weith et al., 2018; Cao et al., 2021). Conceptually, this process is reminiscent of ribosome biogenesis that involves the binding of a transient maturation factor, Rlp24, which needs to be extracted by the p97-related AAA protein Drg1 (Prattes et al., 2022).

SDS22-PP1-I3 disassembly can be reconstituted from purified components, which allowed detailed mechanistic dissection. Of note, p37 with p97 is sufficient to mediate SDS22-PP1-I3 disassembly without ubiquitylation involved (Weith et al., 2018). Cryo-EM revealed that p97-p37 is loaded with the client complex firmly on one of the p97 N-domains in a very different manner from Ufd1-Npl4 (van den Boom et al., 2022) (Figures 4B, C). The SDS22 subunit locks directly into the N-domain groove with a helix in SDS22 (van den Boom et al., 2022). This is surprising and reminiscent of AAA proteins such as VPS4 that, too, directly binds its client through an N-terminal domain (Monroe et al., 2017). The SEP-domain adapter is still essential for client targeting probably by positioning the substrate complex with direct contacts (Kracht et al., 2020). The p37 SHP box binds the same N-domain as SDS22 underneath PP1, while the p37 UBX domain interacts with the adjacent p97 N-domain (van den Boom et al., 2022). Site-specific crosslinks confirmed the position of the linker underneath PP1 and a multivalent interaction of p37 with the SDS22-PP1-I3 complex involving binding of the p37 SEP domain with I3 (Kracht et al., 2020). In the structure, I3 is already inserted in the channel of the spiral-shaped p97 hexamer while the bigger part of I3 is still associated with PP1 (van den Boom et al., 2022) (Figures 4B, C). Together with the firm attachment of the PP1 complex to one of the N-domains, this suggests a hold-and-extract mechanism for disassembly of the SDS22-PP1-I3 complex that may more widely be valid for other disassembly reactions.

A key question is how the direct client I3 is inserted into the p97 channel. Mapping of I3 revealed an internal recognition site in I3 that can also be dominantly crosslinked inside the p97 channel (van den Boom et al., 2021). Consistent with that, blocking both I3 termini by circularization of I3 did not prevent I3 unfolding suggesting that I3 is inserted as a loop (van den Boom et al., 2021). The SEP domain was not resolved in the structure, but also engages in I3 interaction and is essential for I3 unfolding (Kracht et al., 2020). It is therefore possible that the SEP domain has a function in I3 insertion, possibly even melting the I3-PP1 interaction equivalent to the melting of ubiquitin by Npl4 for insertion in the Ufd1-Npl4 pathway.

The other question is why different SEP domain adapters exist in metazoans. Like p37, UBXN2A can target I3 for unfolding *in vitro* raising the question what the functional or regulatory difference between the two adapters is in cells (Kracht et al., 2020). The more divergent UBNX11 does not target the PP1 complex *in vitro*. Surprisingly, despite the sequence similarity to p37, p47 is also not active. However, it can be changed to support PP1 complex disassembly by a transplant of the p37 SHP box-UBX linker to p47 (Kracht et al., 2020). It remains to be determined whether this reflects divergent client specificity of p47 compared to p37 and UBXN2A, or a possible lever for regulation.

## A regulatory role for ubiquitin in Shp1 and p47-directed unfolding?

It is important to note that, whereas p37 and UBXN2A are represented only by a few thousand copies in tissue culture cells, p47 is by far the most abundant p97 adapter (Beck et al., 2011) suggesting that more client proteins and functions of p47 are to be discovered. One such function in yeast is the ubiquitin-directed and Shp1-mediated processing of Def1 for regulation of RNA polymerase II degradation (Lehner et al., 2022). Another function of Cdc48 and Shp1 in yeast is a disassembly reaction associated with regulation of a cullin RING ubiquitin ligase (CRL) (Yen et al., 2012; Lauinger et al., 2020). In unchallenged cells, the transcription factor Met4 is constitutively degraded following ubiquitylation by the CRL Skp1-Cullin-F-box (SCF) with its substrate adapter Met30. In the response to Cd^2+^, however, SCF-Met30 is rapidly disassembled and, thus, Met4 is stabilized to help Cd^2+^ detoxification (Yen et al., 2012).

Importantly, SCF-Met30 disassembly is mediated by Cdc48 and Shp1, and requires autoubiquitylation of Met30 (Yen et al., 2012; Lauinger et al., 2020). Cdc48 can bind Met30 independently of Shp1, raising the possibility that Met30 binds Cdc48 directly similar to SDS22 binding to p97 in PP1 complex disassembly (Lauinger et al., 2020). Nevertheless, Shp1 is essential for SCF-Met30 disassembly, and this activity depends on its SEP domain, but is independent of the UBA domain in Shp1 (Lauinger et al., 2020). Likewise, Shp1 is essential for high temperature resistance of yeast but this does not require the UBA domain (Bohm and Buchberger, 2013). The role of Shp1 is therefore reminiscent of the role of p37 in PP1 complex disassembly, which forms multivalent interactions with the client complex in a ubiquitin-independent manner and binds the client directly through the SEP domain (Kracht et al., 2020). The activity of Cdc48 and Shp1 in SCF-Met30 regulation still needs to be validated and dissected *in vitro*. From the available information, however, and in contrast to Ufd1-Npl4, it seems unlikely that Shp1 uses ubiquitin attached to a client as a tag to initiate client unfolding. Rather, ubiquitylation may regulate the recruitment of the client complex similar to substrate recruitment to the proteasome. Shp1 then engages in direct interaction with the client protein to guide a protein stretch of the client into the pore for subsequent unfolding of the whole protein. This would reconcile the seemingly divergent observations regarding ubiquitin involvement for SEP domain adapters such as p37 and p47, and establish a common mechanism of direct insertion of elements of the client itself independently of whether the SEP domain adapter contains a UBA domain or not. Interestingly, Npl4 has evolutionarily gained the NZF as an additional ubiquitin-binding domain in metazoans (Meyer et al., 2002), which does not seem to be directly involved in the loading of ubiquitin into the D1 domain pore. It could therefore have a similar recruitment function as the UBA of p47 proposed here.

### Do additional targeting pathways exist?

So far, no other p97 cofactor has been rigorously demonstrated to act as a client adapter independently of Ufd1-Npl4 or a SEP-domain adapter. However, cell biological work has identified interesting apparent candidates in specific cellular pathways in need of clarification. One such p97 cofactor complex forms with the cofactors UBXD1 and PLAA that have functionally been linked to sorting of caveolin-1 as well as to the endolysosomal damage response leading to lysophagy (Ritz et al., 2011; Papadopoulos et al., 2017). Of note, and consistent with that, mutations in PLAA cause neurodegeneration associated with endolysosomal sorting defects leading to epileptic encephalopathy in children (Hall et al., 2017). The endolysosome-associated function of PLAA appears to be conserved for the yeast orthologue Ufd3 (also called Doa1) (Ren et al., 2008). However, PLAA and UBXD1 (that only exists in metazoans) bind to the C-terminal tail of p97 close to the D2 domain exit pore. It is therefore difficult to rationalize how clients could be targeted to the D1 domain pore by UBXD1 and PLAA.

UBXN1 (also called SAKS1) is an interesting candidate that contains both a UBA and a UBX domains and has been linked to various protein quality control pathways (Ganji et al., 2018; Feng et al., 2021; Mukkavalli et al., 2021; Mengus et al., 2022). However, an adapter function has not been shown biochemically and UBXN1 apparently lacks a SHP box that seems typical for client adapters. Moreover, UBXN1 has been found associated with Ufd1-Npl4 suggesting that UBXN1 might act as an accessory adapter (Alexandru et al., 2008; Ganji et al., 2018). The DNA-dependent protease SPRTN has been linked to p97 and suggested to also act in recruiting clients (Fielden et al., 2020), but biochemical reconstitution showed the requirement of client ubiquitylation and the Ufd1-Npl4 adapter for p97-mediated unfolding of SPRTN substrates (Kroning et al., 2022). Yet another candidate, the UBXN10 cofactor, is critical for anterograde transport in cilia regulation (Raman et al., 2015). The fact that it lacks a ubiquitin-binding domain suggests a ubiquitin-independent targeting mechanism. Structure prediction, however, does not detect a dedicated domain that could help with client targeting, suggesting that UBXN10 may cooperate with another factor for targeting. Apart from further characterizing potential new client adapters, it will also be interesting to clarify whether cofactors such as DERL1/2 or SVIP may act not only in recruiting p97 to membrane but also directly as accessory adapters to stimulate client unfolding.

## Conclusion remarks and perspective

The dissection of p97 function in diverse pathways over the past 30 years has taught us how important controlled protein unfolding or protein complex disassembly is for cellular homeostasis and regulation. As we now know, the core threading machine is indiscriminate with regard to client proteins. It is therefore important to further understand how client proteins and protein complexes are targeted to p97 for unfolding and disassembly. This certainly includes understanding the role of p47 and determine what its clients are. New approaches to target specific adapter complexes may help (Jiang et al., 2022). Further work should also include examining the host of p97 interacting proteins for activities that assist client targeting or even serve as novel client adapters. Likewise, we need to understand how targeting is regulated, for example through posttranslational modifications other than ubiquitylation including phosphorylation or SUMOylation (Lee et al., 2023). With powerful biochemical unfolding and disassembly assays at hand, there is no excuse to not validate and dissect these activities mechanistically *in vitro*.
